# Genetic Determinants of Cardiovascular Events among Women with Migraine: A Genome-Wide Association Study

**DOI:** 10.1371/journal.pone.0022106

**Published:** 2011-07-14

**Authors:** Markus Schürks, Julie E. Buring, Paul M. Ridker, Daniel I. Chasman, Tobias Kurth

**Affiliations:** 1 Division of Preventive Medicine, Department of Medicine, Brigham and Women's Hospital, Harvard Medical School, Boston, Massachusetts, United States of America; 2 Department of Neurology, University Hospital Essen, Essen, Germany; 3 INSERM Unit 708 – Neuroepidemiology, Paris, France; 4 UPMC Univ Paris 06, F-75005, Paris, France; Julius-Maximilians-Universität Würzburg, Germany

## Abstract

**Background:**

Migraine is associated with an increased risk for cardiovascular disease (CVD). Both migraine and CVD are highly heritable. However, the genetic liability for CVD among migraineurs is unclear.

**Methods:**

We performed a genome-wide association study for incident CVD events during 12 years of follow-up among 5,122 migraineurs participating in the population-based Women's Genome Health Study. Migraine was self-reported and CVD events were confirmed after medical records review. We calculated odds ratios (OR) and 95% confidence intervals (CI) and considered a genome-wide p-value <5×10^−8^ as significant.

**Results:**

Among the 5,122 women with migraine 164 incident CVD events occurred during follow-up. No SNP was associated with major CVD, ischemic stroke, myocardial infarction, or CVD death at the genome-wide level; however, five SNPs showed association with p<5×10^−6^. Among migraineurs with aura rs7698623 in *MEPE* (OR = 6.37; 95% CI 3.15–12.90; p = 2.7×10^−7^) and rs4975709 in *IRX4* (OR = 5.06; 95% CI 2.66–9.62; p = 7.7×10^−7^) appeared to be associated with ischemic stroke, rs2143678 located close to *MDF1* with major CVD (OR = 3.05; 95% CI 1.98–4.69; p = 4.3×10^−7^), and the intergenic rs1406961 with CVD death (OR = 12.33; 95% CI 4.62–32.87; p = 5.2×10^−7^). Further, rs1047964 in *BACE1* appeared to be associated with CVD death among women with any migraine (OR = 4.67; 95% CI 2.53–8.62; p = 8.0×10^−7^).

**Conclusion:**

Our results provide some suggestion for an association of five SNPs with CVD events among women with migraine; none of the results was genome-wide significant. Four associations appeared among migraineurs with aura, two of those with ischemic stroke. Although our population is among the largest with migraine and incident CVD information, these results must be treated with caution, given the limited number of CVD events among women with migraine and the low minor allele frequencies for three of the SNPs. Our results await independent replication and should be considered hypothesis generating for future research.

## Introduction

Migraine is a common and often disabling disorder affecting up to 20% of the general population, women 3–4 times more often than men [1]. Clinically migraine presents with recurrent headache attacks and combinations of gastrointestinal and autonomic nervous system symptoms [2], and up to one third of patients experience transient focal neurological symptoms known as migraine aura.

Migraine pathophysiology is incompletely understood. While migraine is viewed as a primary disorder of the brain, vascular mechanisms are also implicated. For example, endothelial dysfunction and hypercoagulability [3] as well as altered vascular reactivity [4] are found in migraine patients. In addition, the current evidence on the association between migraine and cardiovascular disease (CVD) was summarized in a recent meta-analysis [5], which found a two-fold increased risk for ischemic stroke among migraineurs, in particular those with migraine with aura. Some individual studies suggest that the link between migraine and CVD also extends to myocardial infarction (MI), CVD death, and hemorrhagic stroke [6], [7], [8], [9], [10], [11], [12], [13], [14].

Family and twin studies have shown that genetic factors importantly contribute to the pathophysiology of both migraine and CVD. In addition, with respect to the established association between migraine and CVD, the influence of gene variants has recently been investigated, showing for example a modulatory effect of the *MTHFR* 677C>T [15] and *ACE* D/I [16] polymorphisms among women.

Given the unique physiology among migraineurs, specifically migraineurs with aura, which suggests the potential for a genetic risk of CVD, we sought to investigate the genetic liability for CVD among migraineurs. Such a design eliminates a potential confounding effect of migraine on the association between genetic markers and CVD. This contrasts with a study design comparing migraineurs to non-migraineurs, which would also investigate an interaction between the phenotype migraine and genetic markers on CVD risk.

Hence, we performed a genome-wide association study (GWAS) among women with migraine comparing those who developed a CVD event to those who did not.

## Methods

### Study population

The Women's Health Study (WHS) and its subpopulation with genome-wide genetic data, the Women's Genome Health Study (WGHS), form the base populations for this study, which was approved by the Institutional Review Board of Brigham and Women's Hospital. The WHS was a randomized trial designed to test the benefits and risks of low-dose aspirin and vitamin E in the primary prevention of CVD and cancer [17], [18], in which 39,876 U.S. female health professionals aged ≥45 years and free of major illnesses were enrolled. WHS participants provided extensive information about health and lifestyle at baseline and during ongoing follow-up. Prior to randomization, blood samples were collected from 28,345 participating women, who provided consent for blood based analysis [19]. These women formed the source population for the WGHS.

### Ascertainment of migraine

Participants in WHS were asked on the baseline questionnaire: “Have you ever had migraine headaches?” and “In the past year, have you had migraine headaches?” From this information, women were categorized as “any history of migraine;” “active migraine” (self-reported migraine during the past year); and “prior migraine” (report of ever having had a migraine but none in the year prior to completing the questionnaire). This classification and self-reported migraine have shown good agreement with current International Headache Society (IHS) criteria for migraine [8], [20]. Participants reporting “active migraine” were further asked about specific migraine features, including aura. Responses to the question whether they had an “aura or any indication a migraine is coming,” were used to classify women into active migraine with and without aura. Furthermore, on each of the follow-up questionnaires participants were asked about new occurrences of migraine (“incident migraine”). From this information, we defined two main categories: 1) no migraine (never reported migraine throughout the study), and 2) any migraine (any indication of migraine throughout the study). For additional analyses we also looked at specific migraine features, including migraine aura status, among those reporting “active migraine” at baseline.

### Ascertainment of CVD

During follow-up, women participating in the WHS self-reported cardiovascular events. Medical records were obtained for all events and reviewed by an Endpoints Committee of physicians. Nonfatal stroke was confirmed if the participant had a new focal neurological deficit of sudden or rapid onset that persisted for >24 hours. Major stroke subtype classification (ischemic, hemorrhagic, or unknown) was based on available clinical and diagnostic information with excellent interrater agreement [21]. The occurrence of MI was confirmed if symptoms met World Health Organization criteria and if the event was associated with abnormal levels of cardiac enzymes or abnormal electrocardiograms. Cardiovascular deaths were confirmed by review of autopsy reports, death certificates, medical records, or information obtained from next of kin or family members. We evaluated incident major CVD, a combined endpoint defined as the first of any of these events: nonfatal ischemic stroke, nonfatal MI, or death from ischemic CVD. We also evaluated any first ischemic stroke, any first MI and CVD deaths separately.

### Genotyping

Genotyping in WGHS was performed using the HumanHap300 Duo “+” chips or the combination of the HumanHap300 Duo and iSelect chips (Illumina, San Diego, CA) with the Infinium II protocol and has been described elsewhere [19]. Among individuals with successful genome-wide genotyping, 23,294 were identified as having self-reported European ancestry that could be verified on the basis of multidimensional scaling analysis of identity by state using 1443 ancestry informative markers in PLINK v. 1.07 [22]. In the final dataset there were a total of 339,596 SNPs with a minor allele frequency (MAF) >1%, successful genotyping in >90% of subjects, and deviations from Hardy-Weinberg equilibrium not exceeding P = 10^−6^ in significance.

### Statistical analysis

Age-adjusted logistic regression in PLINK [22] was used to investigate the association between gene variants and CVD events in the WGHS cohort and odds ratios (OR) and 95% confidence intervals (CI) were calculated. In the genome-wide analyses, we assumed an additive relationship between the number of copies of the minor allele of each SNP and the age-adjusted log-odds of CVD events. We considered a threshold of p<5×10^−8^ for genome-wide significance [23]. For sensitivity analysis, we also examined two multivariable-adjusted logistic models for the SNPs implicated in the age-adjusted models. In multivariable-adjusted model 1 we considered the following covariates: age (continuous), history of hypertension (yes, no), low-density lipoprotein (LDL) cholesterol (continuous), high-density lipoprotein (HDL) cholesterol (continuous), menopausal status (yes, no), smoking (never, past, current), and family history of myocardial infarction before age 60 (yes, no). Multivariable-adjusted model 2 was adjusted for the same covariates as model 1 plus the top 10 eigenvectors for sub-European population structure.

We evaluated the associations between SNPs and CVD events among all migraineurs as well as among women with migraine with aura and migraine without aura separately. We further investigated whether the association of previously reported genetic markers for MI [24], ischemic stroke [25], [26], and silent brain infarcts [27] with any of the CVD events is modified by migraine aura status by adding an interaction term between the marker and aura status (yes, no) (SNP*aura) to the logistic models. For candidate gene analysis, SNPs within 25 kb of the transcribed region of each gene were considered (**Table S1**). To estimate the statistical significance of the most strongly associated SNP within the locus accounting for multiple hypothesis testing, we computed a locus-wide effective number of SNPs based on LD relationships in the WGHS and used this number in the Šidák p-value adjustment procedure as described [28].

Additional programming was performed in R. All annotations derive from human genome reference sequence hg18 (NCBI build 36.1), the UCSC Refseq as of October 27, 2008, and the dbSNP database (build 129) as represented by the UCSC database.

## Results

Of the 23,294 women with verified European ancestry, 5122 reported any migraine during the study, 4247 at baseline and 875 during follow-up. Among the women with migraine information from baseline, 3003 reported active migraine and 1244 prior migraine. Further, among women with active migraine 1177 (39.2%) had migraine with aura and 1826 (60.8%) migraine without aura. Information on incident migraine did not allow further classification according to migraine aura status.

Among the 5122 women with any migraine 164 developed a major CVD event during follow-up (73 MIs, 68 ischemic strokes, 35 CVD deaths). The respective numbers of CVD cases for migraine with aura and migraine without aura are: migraine with aura (1177 women)—55 major CVDs, 26 MIs, 21 ischemic strokes, 12 CVD deaths; migraine without aura (1826 women)—38 major CVDs, 18 MIs, 17 ischemic strokes, 7 CVD deaths.

Baseline characteristics of the participating women according to major CVD are summarized in **Table 1**. Women with migraine experiencing major CVD during the study were older, were more likely to have a history of hypertension, and had higher systolic and diastolic blood pressure compared to migraineurs without major CVD. Women with major CVD also had higher LDL and lower HDL cholesterol levels, were more likely to be postmenopausal, and to be current smokers.

**Table 1:** Baseline characteristics of migraineurs according to cardiovascular disease status (n = 5,122).

	Migraineurs without cardiovascular events (n = 4,958)	Migraineurs with cardiovascular events (n = 164)	p-value*
Age, y (SD)	53.3 (6.3)	58.9 (8.3)	9.6×10^−15^
History of hypertension, %	23.5	48.2	8.5×10^−13^
Systolic blood pressure, mmHg (SD)	122.9 (13.2)	131.1 (15.0)	8.6×10^−11^
Diastolic blood pressure, mmHg (SD)	76.9 (9.2)	79.6 (9.4)	3.0×10^−4^
LDL cholesterol, mg/dl (SD)	123.3 (35.1)	129.4 (34.6)	2.5×10^−2^
HDL cholesterol, mg/dl (SD)	53.1 (15.2)	49.3 (16.3)	3.8×10^−3^
Postmenopausal status, %	49.0	67.1	7.5×10^−6^
Smoking, %			
Current	10.6	23.2	
Past	37.1	26.2	
Never	52.4	50.6	7.4×10^−7^
Family history of myocardial infarction before age 60, %	13.9	16.7	0.41

Numbers are means (SD), unless otherwise stated.

*P-value from t-test for continuous data and chi-square test for categorical data.

**Figure 1:**
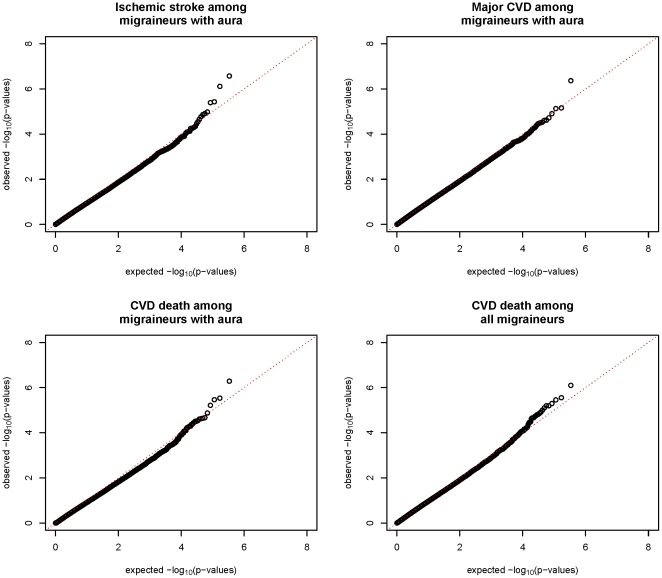
Quantile-quantile plots for the association between SNPs and CVD among women with migraine.

In the genome-wide analysis no SNP reached the conventional significance threshold of p<5×10^−8^ in age-adjusted logistic models assuming an additive relationship between the minor allele dose and log-odds of major CVD, ischemic stroke, MI or CVD death. Nevertheless, investigation of the quantile-quantile plots of probability values for the association between SNPs and CVD events among migraineurs indicated a slight excess of small p-values for some of the associations after controlling for modest inflation of the test statistic (λ_GC_ = 1.03, **Figure 1**). We thus decided to further investigate SNPs suggestive of an association with p-values <5×10^−6^.

Five SNPs on different chromosomes showed associations with CVD events among women with migraine at the p<5×10^−6^ threshold suggesting increased risks for carriers of the minor alleles (**Table 2**). Four of the associations were seen among women with migraine with aura: two SNPs suggested an increased risk for ischemic stroke (rs7698623 [**Figure S1**], odds ratio [OR] = 6.37, 95% confidence interval [CI] 3.15–12.90, p = 2.7×10^−7^; rs4975709 [**Figure S2**], OR = 5.06, 95% CI 2.66–9.62, p = 7.7×10^−7^), one for major CVD (rs2143678 [**Figure S3**], OR = 3.05, 95% CI 1.98–4.69, p = 4.3×10^−7^), and one for CVD death (rs1406961 [**Figure S4**], OR = 12.33, 95% CI 4.62–32.87, p = 5.2×10^−7^). Further, one SNP suggested an increased risk for CVD death among women with any migraine (rs1047964 [**Figure S5**], OR = 4.67, 95% CI 2.53–8.62, p = 8.0×10^−7^). All ORs are large compared with effect estimates for CVD from studies in populations not selected for migraine, which for example report of a doubling of the relative risk for ischemic stroke among migraineurs, particularly migraineurs with aura [5]. Further, our observed ORs need to be interpreted in terms of the rather low minor allele frequencies (MAF) for some of the SNPs (**Table 2**). The significance and magnitude of the associations were very similar in the multivariable-adjusted additive models (**Table 2**).

**Table 2:** Associations between SNPs and CVD events from additive models for p-values <5×10^−6^.

Association	CHR	SNP	BP	Minor/ major allele	MAF	Age-adjusted model		Multivariable-adjusted model 1*		Multivariable-adjusted model 2†	
						OR (95% CI)	p-value	OR (95% CI)	p-value	OR (95% CI)	p-value
MA—ischemic stroke	4	rs7698623	88974851	A/G	0.06	6.37 (3.15–12.90)	2.7×10^−7^	6.31 (3.02–13.17)	9.2×10^−7^	6.48 (2.99–14.03)	2.2×10^−6^
MA—ischemic stroke	5	rs4975709	1930279	C/A	0.24	5.06 (2.66–9.62)	7.7×10^−7^	5.68 (2.88–11.21)	5.3×10^−7^	6.16 (3.03–12.53)	5.2×10^−7^
MA—major CVD	6	rs2143678	41731010	A/C	0.16	3.05 (1.98–4.69)	4.3×10^−7^	3.44 (2.19–5.41)	8.6×10^−8^	3.31 (2.07–5.31)	6.5×10^−7^
Any migraine—CVD death	11	rs1047964	116662102	C/G	0.05	4.67 (2.53–8.62)	8.0×10^−7^	4.64 (2.50–8.61)	1.1×10^−6^	4.71 (2.49–8.90)	1.9×10^−6^
MA—CVD death	20	rs1406961	61366364	A/C	0.09	12.33 (4.62–32.87)	5.2×10^−7^	12.23 (4.46–33.54)	1.2×10^−6^	29.93 (6.44–139.2)	1.5×10^−5^

CHR, chromosome; SNP, single nucleotide polymorphisms; BP, base-pair; MAF, minor allele frequency; OR, odds ratio; CI, confidence interval; MA, migraine with aura; CVD, cardiovascular disease.

*adjusted for: age, history of hypertension, systolic blood pressure, diastolic blood pressure, LDL cholesterol, HDL cholesterol, menopausal status, smoking, and family history of myocardial infarction.

† adjusted for the same covariates as multivariable-adjusted model 1 plus population structure (top 10 eigenvectors for sub-European population structure).

In addition to our model comparing migraineurs with CVD to migraineurs without CVD, we also explored a model comparing migraineurs with CVD to controls free of migraine and CVD (n = 17,247). The top hits were the same for the associations between migraine with aura and ischemic stroke, migraine with aura and major CVD, and any migraine and CVD death (data not shown). For the association between migraine with aura and CVD death the SNP on chromosome 20 (rs1406961) was only the second most significant one, while rs1047964 (see previous paragraph) was the top SNP.

Additional analyses did not indicate that the association between SNPs in genes previously identified as markers for MI, ischemic stroke, and silent brain infarcts (**Table S1**) and any of the CVD events is modified by migraine aura status (data not shown).

## Discussion

The results of this GWAS do not indicate that any of the 339,596 SNPs are associated with CVD events among migraineurs at the genome-wide level. After lowering the significance threshold to 5×10^−6^ there was some suggestion that five SNPs at different loci might be implicated. Two of the SNPs suggested an association with ischemic stroke (rs7698623, rs4975709), one with major CVD (rs2143678), and one with CVD death (rs1406961) among women with migraine with aura. In addition, rs1047964 appeared to be associated with CVD death among women with any migraine. There is no indication that migraine aura status modifies the association between previously described markers for MI, ischemic stroke, and silent brain infarcts with any of the CVD events. Although the WGHS population is among the largest with information about both migraine and incident CVD, our results need to be treated with caution, since the number of CVD events was only modest and the MAFs for three of the SNPs was low.

Migraine, in particular migraine with aura, has been associated with CVD. The evidence was summarized in a recent meta-analysis indicating a two-fold increased risk for ischemic stroke, which only appears for migraine with aura [5]. The evidence is less clear for other CVD events; however, individual studies suggest that this pattern may also extend to MI, CVD death, and hemorrhagic stroke [6], [7], [8], [9], [10],[11],[12],[13],[14]. The association between migraine and ischemic vascular events was independent of many CVD risk factors in many studies [7], [8]. Furthermore, the increased risk of ischemic stroke is more apparent among migraineurs without CVD risk factors [29], [30], [31]. In addition functional [4], cellular [32], and genetic data [33] indicate shared biological vulnerability between vascular disorders and migraine.

Investigating the genetic liability for CVD among migraineurs has only recently begun. Two analyses reported on the influence of single gene variants on the migraine-CVD association and showed a modulatory effect of the *MTHFR* 677C>T [15] and *ACE* D/I [16] polymorphisms among women. The two-fold increased risk of CVD among migraineurs with aura was further raised among those migraineurs with aura also carrying the *MTHFR* 677TT genotype. This was only seen for ischemic stroke and not for MI. With regard to the *ACE* D/I polymorphism, only migraineurs with aura carrying the DD/DI genotype seemed to be at increased risk for ischemic stroke and MI, but not carriers of the II genotype.

The approach of our study is novel in two different ways. First, we compared migraineurs with CVD events to migraineurs without CVD events in contrast to previous studies comparing migraineurs to non-migraineurs. This design eliminates a potential confounding effect of migraine with gene variants on CVD events and specifically addresses the question which gene variants are associated with CVD events among migraineurs. Second, our study investigates the effect of genetic markers among migraineurs on their risk for CVD events from a genome-wide perspective.

None of the investigated SNPs was significantly associated with CVD events among migraineurs at a genome-wide level. However, our analyses provide some indication of an association of five genetic variants with CVD events at a suggestive significance threshold. In addition, our results—similar to previous non-genetic studies—support the concept that the risk for CVD among migraineurs is not strongly confounded by classical CVD risk factors. First, results from the multivariable-adjusted models are very similar to age-adjusted models (**Table 2**), although CVD risk factors are more prevalent among migraineurs with CVD events than among those without CVD events (**Table 1**). This is likely due to a co-linearity between age and classical CVD risk factors. Second, none of the five implicated genetic variants has previously been reported to be associated with CVD risk factors or CVD events.

The potential function for the five variants with regard to an increased CVD risk among migraineurs is unclear. A summary of the known functional characteristics of these variants is presented in **Table 3**. However, it is important to note, that four of the SNPs were identified for the association between migraine with aura and CVD events and two of those specifically for the association with ischemic stroke. This agrees with data from observational studies suggesting a particularly strong link between migraine with aura and ischemic stroke [5].

**Table 3:** Functional characteristics of genetic variants implicated in the association with CVD among migraineurs (with p<5×10^−6^).

Chromosomal region	SNP	Gene (coded protein)	variant location	Tissue Expression	Gene Function	genes ±50000 bp around SNP	distance SNP-gene	Tissue Expression	Function
4q22.1	rs7698623	MEPE (matrix extracellular phosphoglycoprotein)	intron	Nucleus subthalamicus, trigeminal ganglion, superior cervical ganglion, skin	Bone formation and tumors causing osteomalacia [36]	IBSP (integrin-binding sialoprotein precursor)	22227	Temporal lobe, parietal lobe, trigeminal ganglion, skin, skeletal muscle	Major structural protein of bone matrix [37]
5p15.33	rs4975709	IRX4 (iroquois homeobox protein 4)	near 3′UTR of IRX4	prostate, heart, lymphoblasts, spinal cord	Mediating ventricular differentiation during cardiac development [38]	None	NA	NA	NA
6p21.1	rs2143678	none	NA	NA	NA	MDF1 (MyoD family inhibitor)	1051	Bronchial epithelial cells, tongue, skeletal muscle	Negatively regulates subset of helix-loop-helix proteins, thus influencing trophoblast and chrondrogenic differentiation [39]
						TFEB (transcription factor EB)	-28683	heart, leukocytes	Involved in lysosomal degradation [40]
11q23.3	rs1047964	BACE1 (beta-site APP-cleaving enzyme 1 isoform A)	3′UTR	CNS	Implicated in generation of A-beta peptides in Alzheimer's disease [41]	RNF 214 (ring finger protein 214)	489	leukocytes	unknown
20q13.33	rs1406961	none				ARF1GAP (ADP-ribosylation factor GTPase activating protein 1)	-8245	leukocytes	Associates with Golgi apparatus and interacts with ADP-ribosylation factor 1 [42]
						HRIHFB2281 (highly similar to ADP-ribosylation factor GTPase-activating protein 1)	-20046	leukocytes	unknown
						NKAIN4 (Na^+^/K^+^ transporting ATPase interacting 4)	10030	CNS, lymphoblasts, kidney	unknown
						BIRC7 (Baculoviral IAP repeat-containing protein 7)	24065	leukocytes	Inhibitor of apoptosis [43]

Our study has several strengths, including CVD events being verified by an endpoints committee of physicians, detailed information on many potential CVD risk factors, and genetic information from 339,596 SNPs covering the whole genome. In addition, the homogenous nature of the cohort, consisting only of white women aged ≥45 years, may reduce confounding. For example, while there is no reason to assume that migraine pathophysiology is different between women and men, the phenotypic expression among migraineurs may differ by gender and age. This is suggested, for example, by data showing that the migraine-ischemic stroke association is greater among younger women than older women [5], which may depend on a changing pattern in cardiovascular risk profile [30].

However, several limitations should be considered. First, and most importantly, data to directly replicate our results are not available to verify our results or perform a meta-analysis. However, we believe that this should not hold back research, since our results may stimulate new research ideas and accelerate ongoing research. Second, we cannot exclude the possibility of spurious associations. The number of women with migraine experiencing a CVD event was limited (n = 164) and the odds ratios for the five SNPs are rather large with effect sizes between 3 and 12 compared to usually much smaller odds ratios found in association studies for complex and heterogeneous disorder. For rs7698623, rs1047964, and rs1406961 this may also in part be due to the low MAFs (all <10%; **Table 2**). Hence, our data should be interpreted with caution. Third, migraine and aura status were self-reported and were not classified according to strict IHS criteria [2]. Although previous reports from the WHS have shown good agreement of our classification with modified IHS criteria from 1988 [8], and despite excellent agreement between self-reported migraine and migraine classification based on IHS criteria from 2004 in the WHS [20], non-differential misclassification is possible, which may in part explain some of our null findings. However, the prevalence of migraine (18.3%) and migraine aura (39.5% of migraineurs) is similar to those reported in other population-based studies [34], [35]. Finally, we only considered an additive genetic model of transmission. However, the vast majority of gene-phenotype associations follow an additive model, which would also have sufficient power to capture dominant modes of transmission. While recessive modes of transmission may be missed, we consider this a minor disadvantage because they are rare and mostly relevant for monogenic disorders.

Our results demand additional epidemiological studies and basic science research and should be considered hypothesis generating at present. First, large well-defined cohorts are needed with standardized information on migraine and aura status, gender, ethnicity, CVD risk factors, and other medical as well as genetic information, to verify the potential role of the gene variants for the CVD risk among migraineurs. In addition, further insight into the biological function of these genes may advance the understanding of the migraine-CVD link.

## Supporting Information

Figure S1 **Regional plot for associations near rs7698623 among migraineurs with aura with ischemic stroke.**
(TIF)

Figure S2 **Regional plot for associations near rs4975709 among migraineurs with aura with ischemic stroke.**
(TIF)

Figure S3 **Regional plot for associations near rs2143678 among migraineurs with aura with major CVD.**
(TIF)

Figure S4 **Regional plot for associations near rs1406961 among women with any migraine with CVD death.**
(TIF)

Figure S5 **Regional plot for associations near rs1047964 among migraineurs with aura with CVD death.**
(TIF)

Table S1 Candidate genes of SNPs investigated for interaction with migraine aura status on CVD risk.
(DOC)
