# Supplementary figures and images for: Genetic Determinants of Cardiovascular Events among Women with Migraine: A Genome-Wide Association Study
Source: PLoS One. 2011 Jul 14;6(7):e22106. doi: 10.1371/journal.pone.0022106 (PMC3136515; doi:10.1371/journal.pone.0022106)

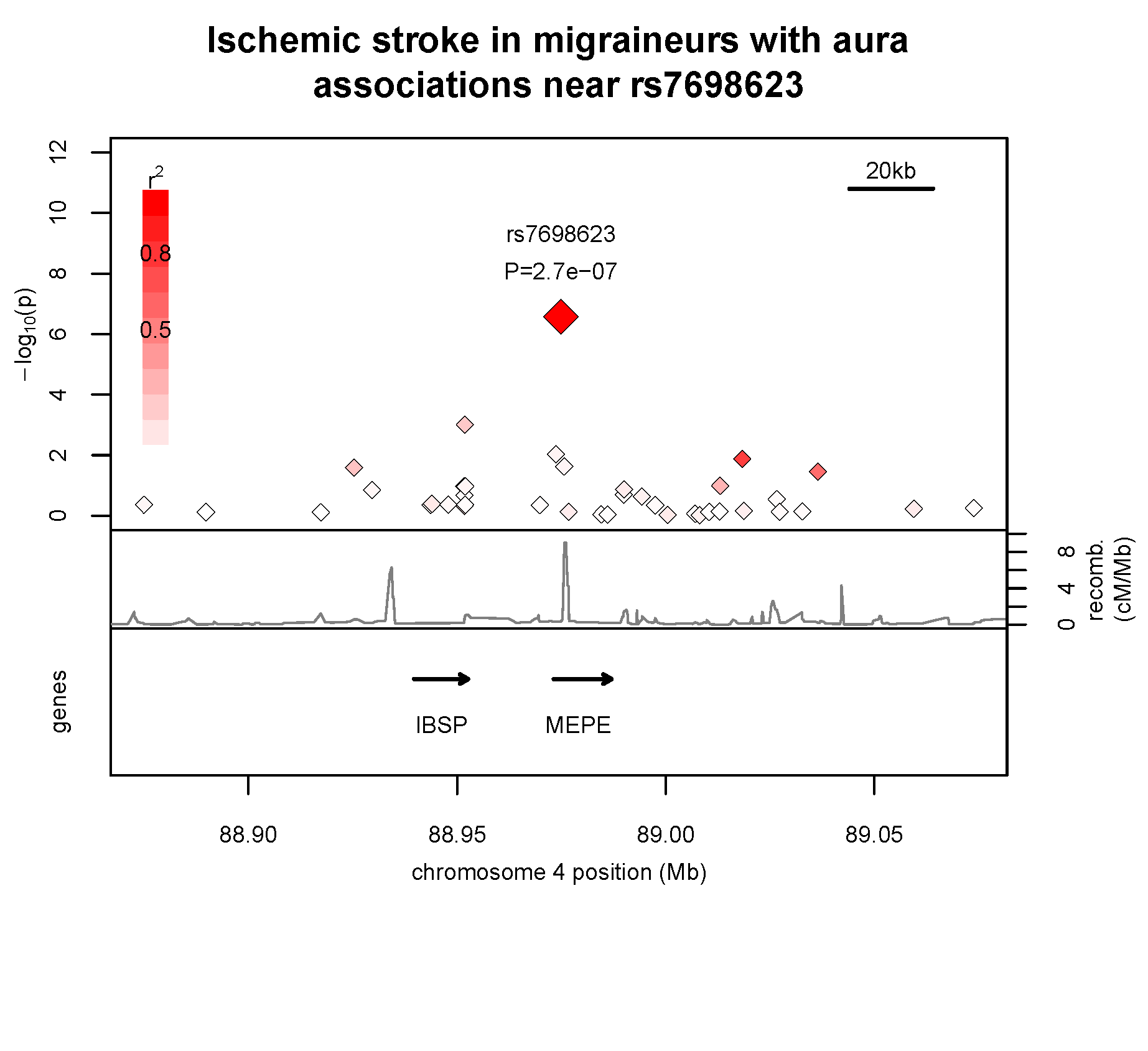

Supplement: Figure S1 — Regional plot for associations near rs7698623 among migraineurs with aura with ischemic stroke. (TIF) [file pone.0022106.s001.tif]

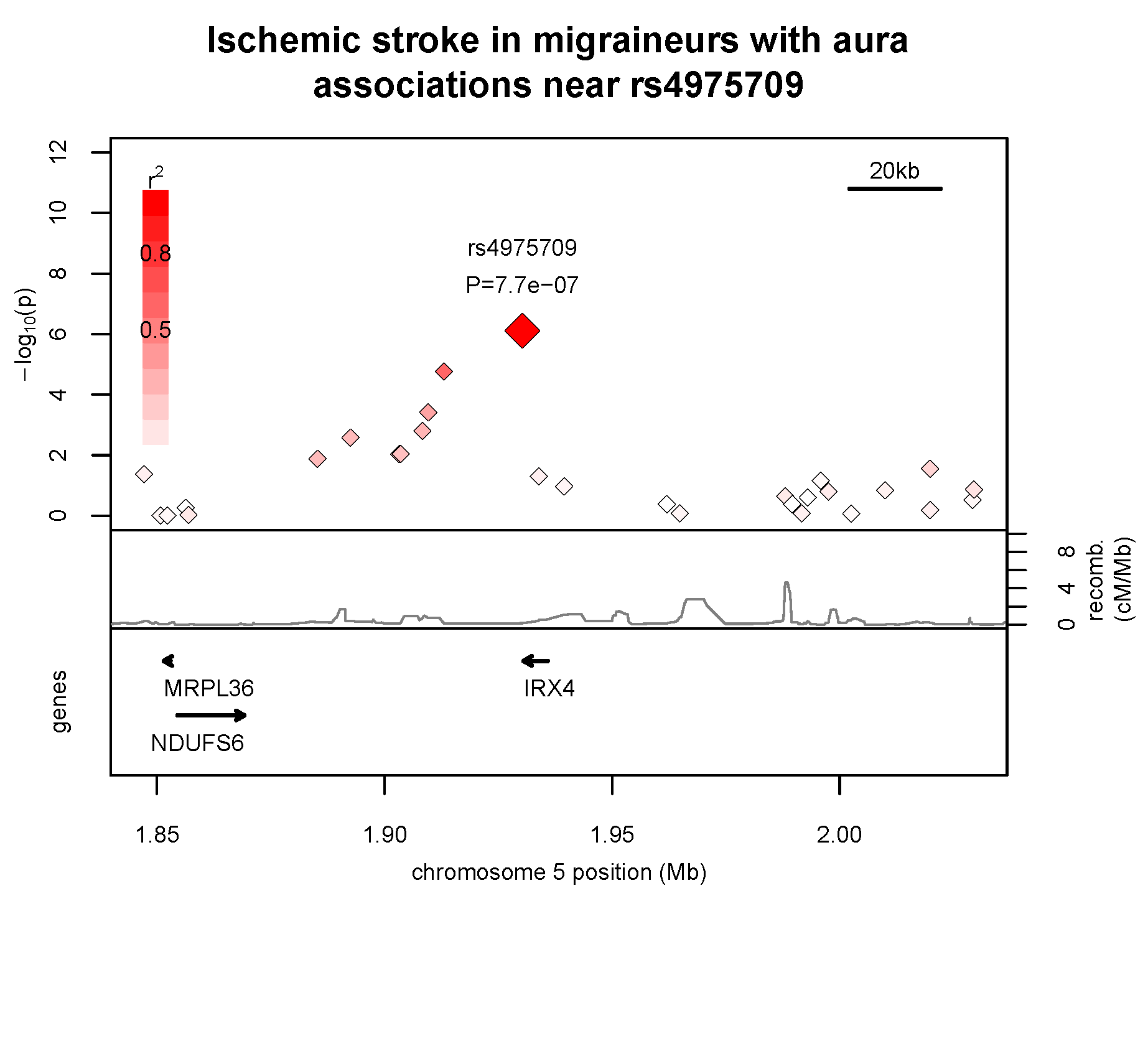

Supplement: Figure S2 — Regional plot for associations near rs4975709 among migraineurs with aura with ischemic stroke. (TIF) [file pone.0022106.s002.tif]

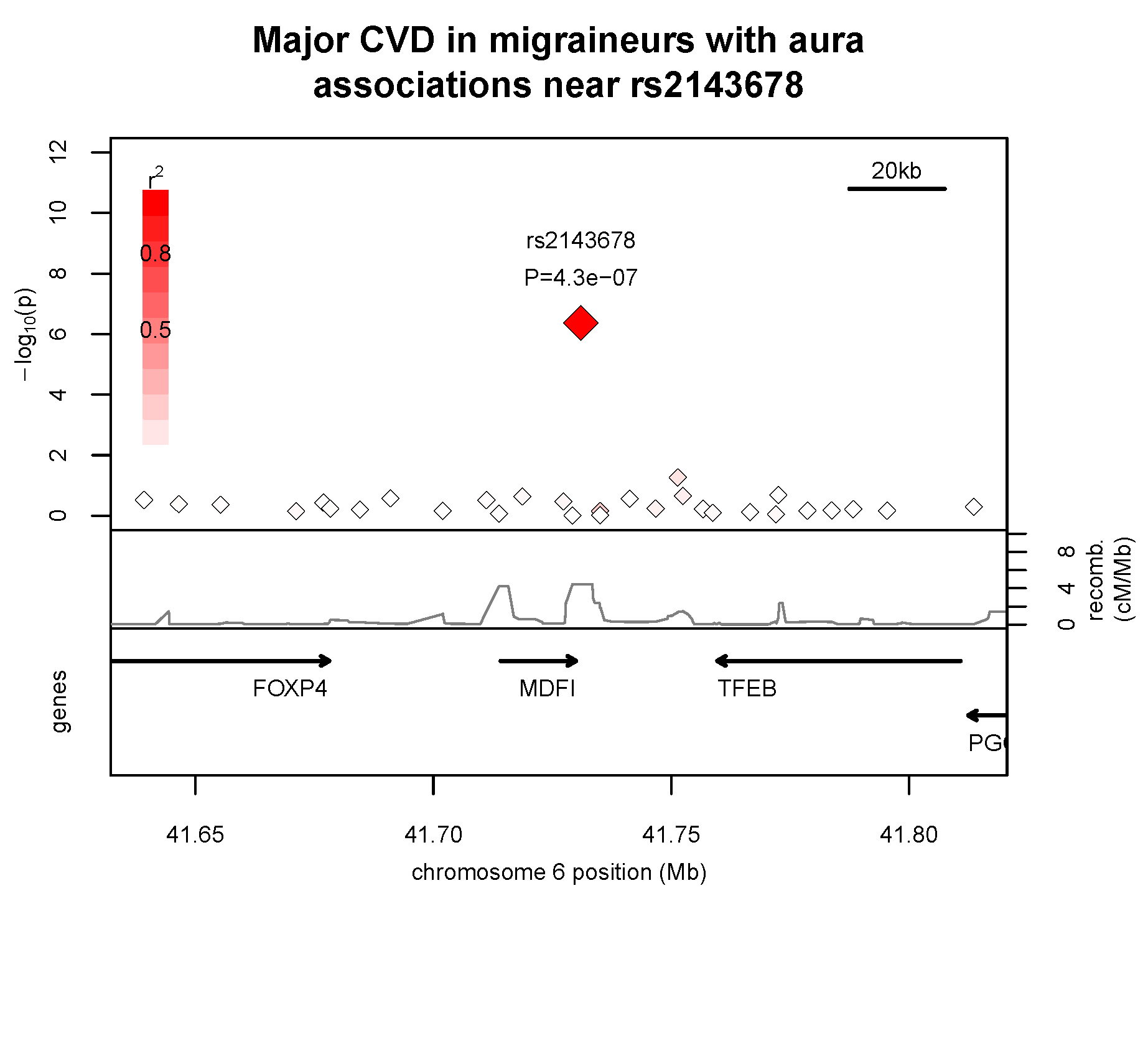

Supplement: Figure S3 — Regional plot for associations near rs2143678 among migraineurs with aura with major CVD. (TIF) [file pone.0022106.s003.tif]

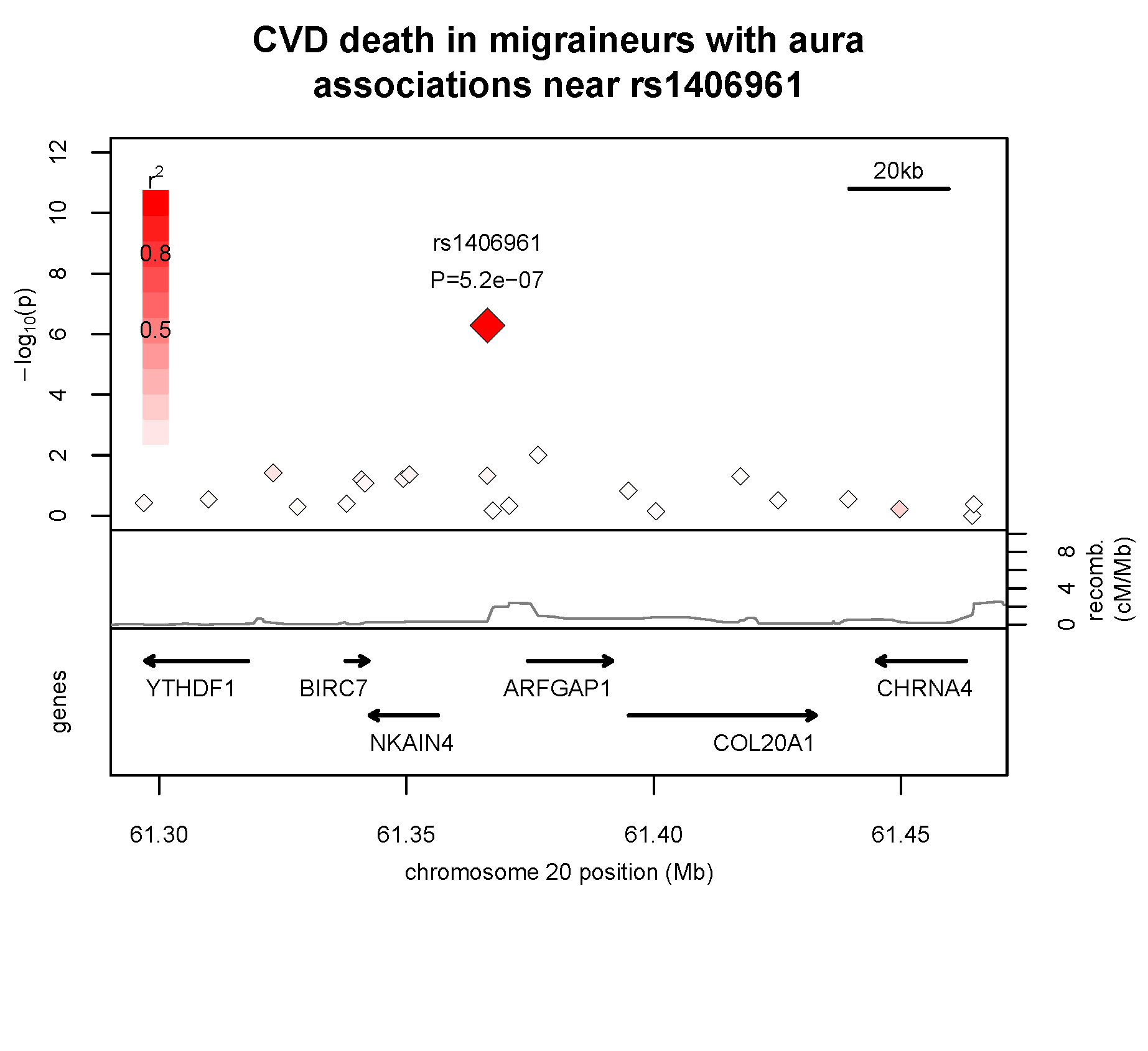

Supplement: Figure S4 — Regional plot for associations near rs1406961 among women with any migraine with CVD death. (TIF) [file pone.0022106.s004.tif]

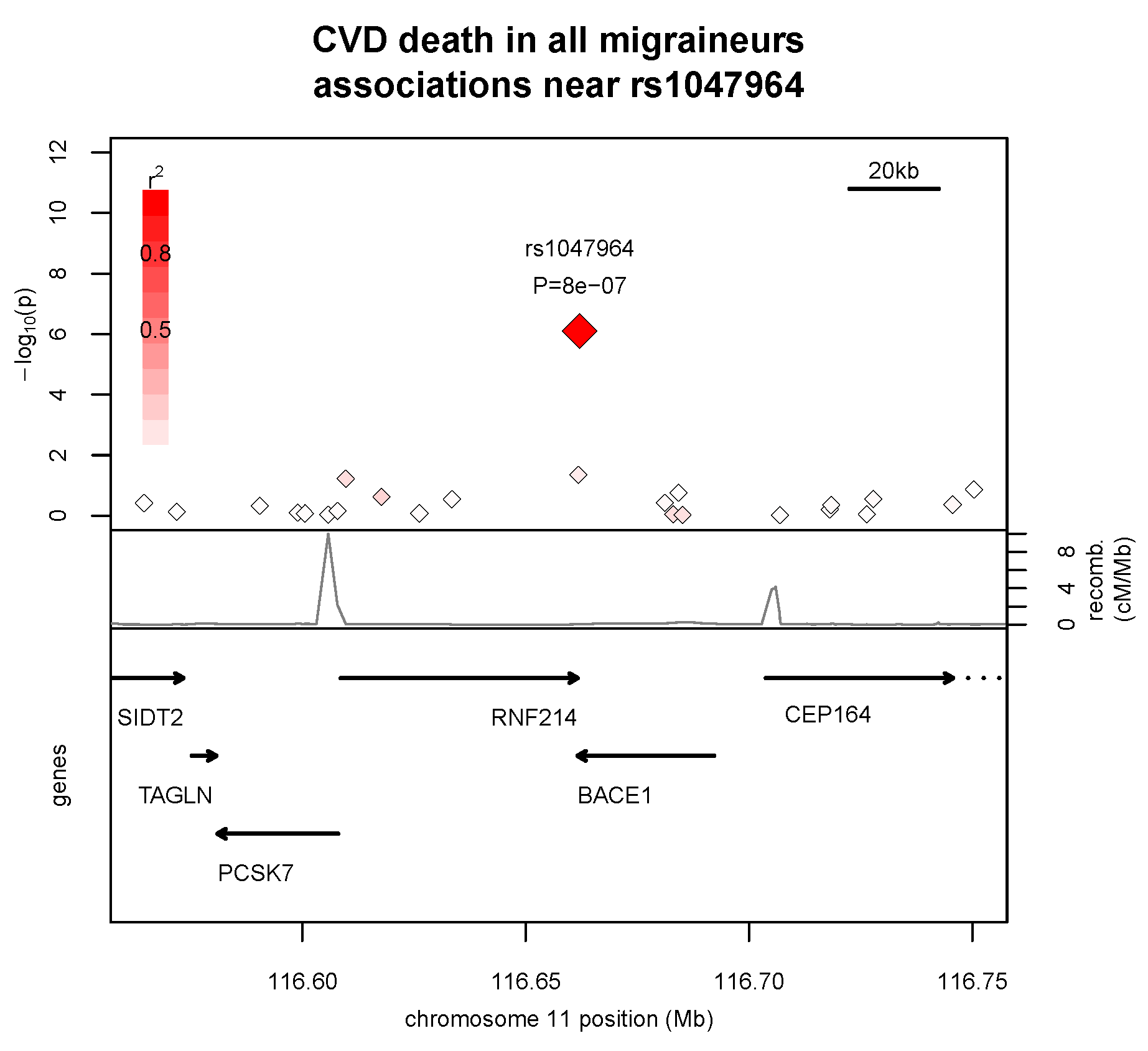

Supplement: Figure S5 — Regional plot for associations near rs1047964 among migraineurs with aura with CVD death. (TIF) [file pone.0022106.s005.tif]
