# Supplementary material for: Genetic Determinants of Cardiovascular Events among Women with Migraine: A Genome-Wide Association Study
Source: PLoS One. 2011 Jul 14;6(7):e22106. doi: 10.1371/journal.pone.0022106 (PMC3136515; doi:10.1371/journal.pone.0022106)
Supplement: Table S1 — Candidate genes of SNPs investigated for interaction with migraine aura status on CVD risk. (DOC) [file pone.0022106.s006.doc]

***Table S1***: Candidate genes of SNPs investigated for interaction with migraine aura status on CVD risk

| ACE |
| --- |
| ALOX5AP |
| CCDC113 |
| CDKN2A |
| CDKN2B |
| CELSR1 |
| CELSR2 |
| CRABP1 |
| CXCL12 |
| EIF4G2 |
| ESRRG |
| F5 |
| FLRT3 |
| GALNTL4 |
| GENE |
| GP1BA |
| GZMB |
| IREB2 |
| KCNE2 |
| KLKBL4 |
| LDLR |
| MACROD2 |
| MRPS6 |
| MTHFD1L |
| MTHFR |
| NINJ2 |
| NOS3 |
| PCSK9 |
| PDE4D |
| PDE6A |
| PHACTR1 |
| PITX2 |
| PLCL1 |
| PPARGC1B |
| PRKCH |
| PSRC1 |
| RAC2 |
| SEC11C |
| SLC5A3 |
| SMAD3 |
| SORT1 |
| SOX17 |
| SSTR3 |
| STXBP6 |
| USH2A |
| WDR12 |
| ZFHX3 |
| ZNF532 |
